# Implications of screening and childcare exclusion policies for children with Shiga-toxin producing *Escherichia coli* infections: lessons learned from an outbreak in a daycare centre, Norway, 2012

**DOI:** 10.1186/s12879-014-0673-2

**Published:** 2014-12-18

**Authors:** Emily MacDonald, Per Kjetil Dalane, Preben Aavitsland, Lin Thorstensen Brandal, Astrid Louise Wester, Line Vold

**Affiliations:** Department of Infectious Disease Epidemiology, Norwegian Institute of Public Health, Nydalen, Oslo, NO-0403 Norway; European Programme for Intervention Epidemiology Training (EPIET), European Centre for Disease Prevention and Control, Stockholm, Sweden; Municipality of Vennesla, Vennesla, 4701 Norway; Epidemi, Lasarettet, Odderøya, Kristiansand, 4610 Norway; Department of Foodborne Infections, Norwegian Institute of Public Health, Nydalen, Oslo, NO-0403 Norway

**Keywords:** Escherichia coli, Shiga-toxin producing Escherichia coli, Enteropathogenic Escherichia coli, Disease outbreaks, Child, Child day care centers, Nurseries

## Abstract

**Background:**

In Norway, it is recommended that children with Shiga-Toxin producing *Escherichia coli* (STEC) infections are excluded from daycare centers until up to five consecutive negative stool cultures are obtained. Children with gastrointestinal illness of unknown etiology are asked to remain home for 48 hours after symptoms subside. On 16 October 2012, two cases of STEC infection were reported from a daycare center, where other children were also symptomatic. Local health authorities temporarily closed the daycare center and all children and staff were screened for pathogenic *E. coli*. We present the results of the outbreak investigation in order to discuss the implications of screening and the exclusion policies for children attending daycare in Norway.

**Methods:**

Stool specimens for all children (n = 91) and employees at the daycare center (n = 40) were tested for pathogenic *E. coli*. Information on demographics, symptoms and potential exposures was collected from parents through trawling interviews and a web-based questionnaire. Cases were monitored to determine the duration of shedding and the resulting exclusion period from daycare.

**Results:**

We identified five children with *stx1-* and *eae*-positive STEC O103:H2 infections, and one staff member and one child with STEC O91:H- infections. Three additional children who tested positive for *stx1* and *eae* genes were considered probable STEC cases. Three cases were asymptomatic. Median length of time of exclusion from daycare for STEC cases was 53 days (range 9 days – 108 days). Survey responses for 75 children revealed mild gastrointestinal symptoms among both children with STEC infections and children with negative microbiological results. There was no evidence of common exposures; person-to-person transmission was likely.

**Conclusions:**

The results of screening indicate that *E. coli* infections can spread in daycare centres, reflected in the proportion of children with STEC and EPEC infections. While screening can identify asymptomatic cases, the implications should be carefully considered as it can produce unanticipated results and have significant socioeconomic consequences. Daycare exclusion policies should be reviewed to address the management of prolonged asymptomatic shedders.

**Electronic supplementary material:**

The online version of this article (doi:10.1186/s12879-014-0673-2) contains supplementary material, which is available to authorized users.

## Background

Shiga-toxin producing *Escherichia coli* (STEC) is a leading cause of gastrointestinal illness, ranging in severity from mild diarrhea to hemorrhagic colitis. Complications include hemolytic uremic syndrome (HUS), which can lead to death [1]. STEC can be transmitted to humans through consumption of contaminated food or water, through direct contact with carrier animals or their fecal material, or through person-to-person transmission [2]. Outbreaks of STEC infections in childcare facilities [3]-[7] pose a particular threat to public health, as children under 5 years old are most frequently diagnosed with infection and are at greatest risk of developing HUS [2]. The majority of STEC carry *eae*, a gene encoding the attaching and effacing (A/E) protein intimin, which is important for the attachment of the bacterium to the epithelial cells in the colon. STEC produce one or both of Shiga toxin 1 (Stx1) and Shiga toxin 2 (Stx2). Epidemiological studies have shown that STEC isolates producing Stx2, or both Stx1 and Stx2, are more commonly associated with HUS than isolates producing only Stx1 [8].

STEC has been notifiable in Norway since 1995, and following an outbreak of STEC O103:H25 in 2006, all cases must be reported within 24 hours of suspicion to the National Institute of Public Health (NIPH). Between 1994 and 2011, 434 cases of STEC were notified in Norway, of which 38% (165 cases) were hospitalized and 12% (53 cases) developed HUS [9]. Although the greatest period of transmissibility is likely to be when cases are symptomatic, post-symptomatic shedding can also lead to community spread of infection [1]. The duration of shedding of STEC after symptoms have ceased varies and only limited information is available for non-O157 strains [9]. However, the median duration of shedding reported from previous outbreaks in childcare facilities has been found to be between 20 and 50 days [5],[7],[10]. A review of 90 outbreaks of STEC O157 showed that higher rates of secondary transmission were found in outbreaks where the median age of cases was <6 years and in outbreaks occurring through person to person spread in childcare facilities [11]. This may be related to close contact of children with immature immune systems and underdeveloped personal hygiene skills, in addition to the long duration of shedding.

In order to prevent transmission of the infection, the NIPH recommends different control measures, depending on the clinical presentation, epidemiological context, and virulence profile and STEC serogroup of the cases. These include stringent daycare exclusion policies for children diagnosed with STEC infections [12]. Children who have tested positive for STEC should remain home until they have had a specific number of negative control tests, taken at least 24 hours apart. This applies to both culture-confirmed cases and cases in which the *stx* and *eae* genes only are detected in faecal samples. If a case is reported that is *stx*- and *eae*-gene positive, but culture negative, control measures are implemented, pending further test results. The number of consecutive negative control tests required is dependent on the severity of clinical presentation, the virulence of the STEC strain and the likelihood of transmission. Children diagnosed with *stx2*-positive STEC or an STEC serogroup that has been frequently associated with HUS should have five consecutive negative results before returning to daycare, regardless of symptom severity. Children with uncomplicated diarrhea with only *stx1*-positive STEC should have three negative control tests before returning to daycare, unless the *stx1*-positive serotype has been previously associated with HUS, in which case five consecutive negative tests are required. All children with STEC infection with severe clinical presentation, such as bloody diarrhoea or HUS, require five negative control tests.

The NIPH recommends that when a case of STEC infection occurs in a daycare setting, other children in the daycare with gastrointestinal symptoms should also be tested. Although it is generally not advised to test asymptomatic children, screening of all children in a daycare center can be considered in situations where person-to-person transmission within a daycare facility is suspected, at the request of local public health authorities. In addition, any child with gastrointestinal symptoms of unknown etiology is asked to remain home while symptomatic and to not return to daycare for 48 hours after symptoms have subsided.

On Tuesday 16 October, the NIPH was notified by the Municipal Health Officer of two cases of *stx1*- and *eae*-positive STEC O103:H2 infection among children attending a daycare center in southern Norway. In addition, the daycare reported that diarrheal illness had affected a considerable number of children attending the daycare throughout September and October. As STEC O103:H2 is known to have previously caused bloody diarrhea and, very rarely, HUS [13],[14], local health authorities and the daycare staff elected to close the centre for extensive cleaning on 17 October. As more STEC cases were suspected, all children were asked to have stool samples tested for STEC prior to returning to the daycare centre. The daycare reopened on 22 October for all children who had negative test results for STEC. An outbreak investigation was initiated in collaboration with local health and food safety authorities in order to determine the extent of the outbreak, identify sources of infection and estimate the duration of shedding in order to implement control measures and minimize the impact in future outbreaks in daycare centres. We present the results of the outbreak investigation and discuss the implications of screening and the exclusion policies for children attending daycare in Norway.

## Methods

### Description of the daycare center

The daycare center is a new and modern daycare centre with 40 staff and capacity for approximately 90 children, distributed among five sections. Two sections (A and B) are for young children aged three and under. Two sections (C and D) are for older children aged three to five. The fifth section (E) is an ‘outdoor’ group for children aged four to five, who spend most of the day outside in the woods.

### Case definition and identification

All family doctors in the outbreak municipality are located in the community health center and were alerted to the outbreak situation. The local clinical microbiological laboratory was also informed of the outbreak.

The following laboratory-based case definitions were developed for this outbreak:

- *Probable case:* Any child attending the daycare with *a preliminary stx-gene finding only* in a stool sample between 1 September and 31 October, 2012.
- *Confirmed case*: Any child attending the daycare with STEC infection confirmed by the National Reference Laboratory between 1 September and 31 October, 2012.
  Definitions of probable and confirmed case also included asymptomatic cases identified through the screening.

### Microbiological investigation

The two initial STEC cases were tested for standard gastrointestinal pathogens (*Salmonella*, *Campylobacter*, *Yersinia* and *Shigella*) as well as STEC. Following the decision to screen all children, stool samples were collected at the community health center and then sent to the local laboratory for analysis using a PCR-method detecting *eae, stx1* and *stx2*. Specimens collected through screening were not tested for pathogens other than *E. coli*. Preliminary test results for the children and employees of the daycare centre were provided to the Municipal Health Officer, who then forwarded them directly to the NIPH. Positive results were also reported by the laboratory to the NIPH via the Norwegian Surveillance System for Communicable Disease (MSIS). All *eae-* and *stx-*positive *E. coli* isolates were sent to the National Reference Laboratory (NRL) at NIPH for confirmation, O:H serotyping, *stx* subtyping [15], and genotyping by multiple-locus variable number of tandem repeat analysis (MLVA) [16]. For cases who tested positive for STEC, follow-up tests were taken at a minimum interval of 24 hours until five consecutive specimens were obtained. Duration of shedding was calculated as the period between symptom onset (or date of testing for asymptomatic cases) and the date of the first negative control test. Duration of exclusion from daycare was calculated as the period of symptom onset (or date of testing for asymptomatic cases) to the date of the last required control test.

### Epidemiological investigation

The local branch of the Norwegian Food Safety Authority (NFSA) conducted standardized trawling interviews with the parents of probable and confirmed cases in order to generate hypotheses concerning possible common sources of infection. These questionnaires were used to collect detailed information on demographic information, clinical information including type and duration of symptoms, food consumption, animal contact and environmental exposures.

In addition, a descriptive cohort investigation of all children attending the daycare was conducted in order to investigate the occurrence of diarrhea reported by parents and identify possible exposures for the period 1 September – 17 October, 2012. For the purposes of this study, no specific definition of diarrhea was provided to parents. Due to the age of the cases, many were still wearing diapers and the frequency of diarrhea using a conventional definition such as more than three loose stools within 24 hours may have been difficult to establish. Parents were asked to specify whether their child had looser stools than normal, more frequent stools than normal and/or diarrhea. Parents were provided a link to a web-based questionnaire using Questback, which was made available October 29 to November 7 (Additional file 1). Reminders to respond to the questionnaire were sent by email on 2 November and by SMS on 6 November. The link to the questionnaire was accompanied by a letter stating that participation was voluntary and the confidentiality of all patients would be maintained. Response to the questionnaire was considered consent for participation. As the survey and other data collection described in this report were part of a public health investigation of an acute event, clearance from an ethical review board for research was not necessary, as permitted by Norwegian legislation.

A descriptive analysis of the results of the questionnaire was conducted using Excel 2010.

### Environmental investigation

The NFSA visited the daycare centre on 16 October in order to inspect the facility. In particular, food hygiene practices and food handling procedures, cleaning routines, kitchen staff hygiene measures, and organization were inspected. Food handlers provided the NFSA with copies of menus for the period prior to the outbreak. An external consultant also inspected the washroom and diaper-changing areas.

## Results

### Description of STEC cases

All 91 children attending the daycare centre during the study period submitted a stool sample prior to returning to the daycare centre. Six children were classified as confirmed cases: specimens from five children were positive for *E. coli* O103:H2, *eae* and *stx1a* with identical or similar MLVA profiles (one locus difference), while one child had a specimen that was positive for STEC O91:H- and was *stx1a-* and *stx2b-*positive. This corresponds to an overall attack rate of 7%. Three additional children tested positive for *eae* and *stx1* at the local laboratory in mixed fecal culture but the NRL was unable to isolate STEC for two of the children and the third child did not have a specimen provided to the NRL. These cases were classified as probable cases. The median age of all STEC cases was 2 years (range 1 to 4 years) and only one case was female. The most commonly reported symptom was diarrhea (6/9 cases), with several cases reporting fever (4/9 cases). No cases developed bloody diarrhea or severe complications. Three cases were asymptomatic and only identified through screening. Onset of symptoms occurred between 8 September and 16 October 2013. The probable and confirmed cases were identified among children attending three different daycare sections (Table 1). Attack rates for probable and confirmed cases among the daycare groups ranged from 0% in Groups C and E to 21% in Group A.

**Table 1:** **Attack rates for probable and confirmed cases by daycare group, September 1 – October 17, 2012**

Daycare section	No. of children	Probable cases	Confirmed cases	Attack rate (probable + confirmed)	Attack rate (confirmed)
**A** (≤ 3 years)	14	1	2	21%	14%
**B** (≤ 3 years)	15	0	2	13%	13%
**C** (3-5 years)	24	0	0	0%	0%
**D** (3-5 years)	25	2	2	16%	8%
**E** (4-5 years*)	13	0	0	0%	0%
**Total**	**91**	**3**	**6**	**10**%	**7**%

*Outdoor group.

**Figure 1:**
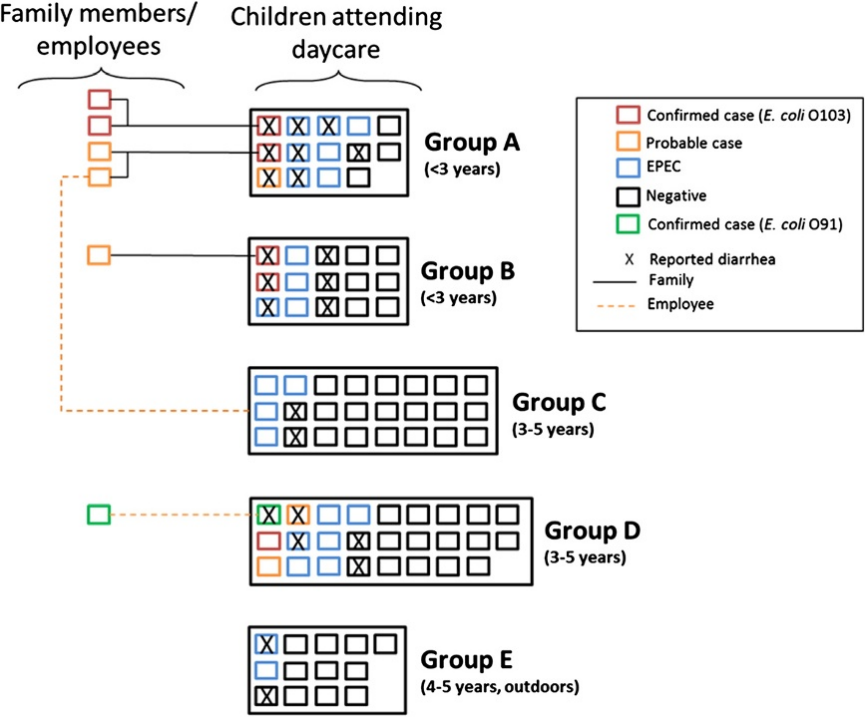
**Parent-reported diarrhea and test results for pathogenic** ***E. coli*** **for all children at the daycare by group, October 2012, Norway.** *Each square represents one person.

In addition to the nine cases in children, there was one confirmed case among staff members (STEC O91:H-, *stx1a*), who was asymptomatic and identified through screening, as well as two confirmed cases (both *E. coli* O103:H2, *stx1a* and *eae*) and three probable cases among family members of three children, all of whom had mild symptoms. The staff member worked primarily with Group D while the affected family members were linked to children in Groups A and B (Figure 1). Furthermore, screening identified twenty-nine children in the daycare with *eae* positivity only, indicative of enteropathogenic *Escherichia coli* (EPEC). The specimens were not further processed in order to isolate the *eae*-positive strains and were not submitted to the NRL for further analysis. These children were not required to submit follow-up control tests.

### Epidemiological investigation

The parents of eight children with probable or confirmed STEC infections were interviewed by employees of the NFSA. In addition, questionnaire responses were submitted for 75 of 91 children attending the daycare (82%). Response rate varied between the different daycare groups, ranging from 69% to 96%. A review of the trawling questionnaires and the descriptive cohort study did not reveal any common sources of infection among cases related to food items, water sources, contact with animals or travel history among children or their parents.

Twenty-two children (30%) had parent-reported diarrhea (looser stools than normal, more frequent stools than normal and/or diarrhea) at least once between 1 September and 31 October, 2012. Six parents characterized their child’s symptoms as watery diarrhea but none reported bloody diarrhea. The duration of symptoms ranged from half a day to over four weeks. Groups A, B and D had relatively more children with diarrhea and EPEC cases than the other groups, as well as confirmed epidemiological links with cases in household members and employees (Figure 1). Among Group E, the ‘outdoor’ group, no cases of STEC and few cases of diarrhoea were identified.

Nine parents (12%) responded that it was difficult to determine whether their child had diarrhea. Parents also had differing criteria for keeping their child home from daycare prior to the outbreak. While almost all parents answered that they would keep their child home if they had a fever or seemed ill in addition to experiencing gastrointestinal symptoms, only 65% would keep their child home based on the conventional definition of diarrhea (more than 3 loose stools within 24 hours). Only 33% would keep their child home based solely on a change in stool consistency or frequency.

### Duration of shedding in STEC cases

**Figure 2:**
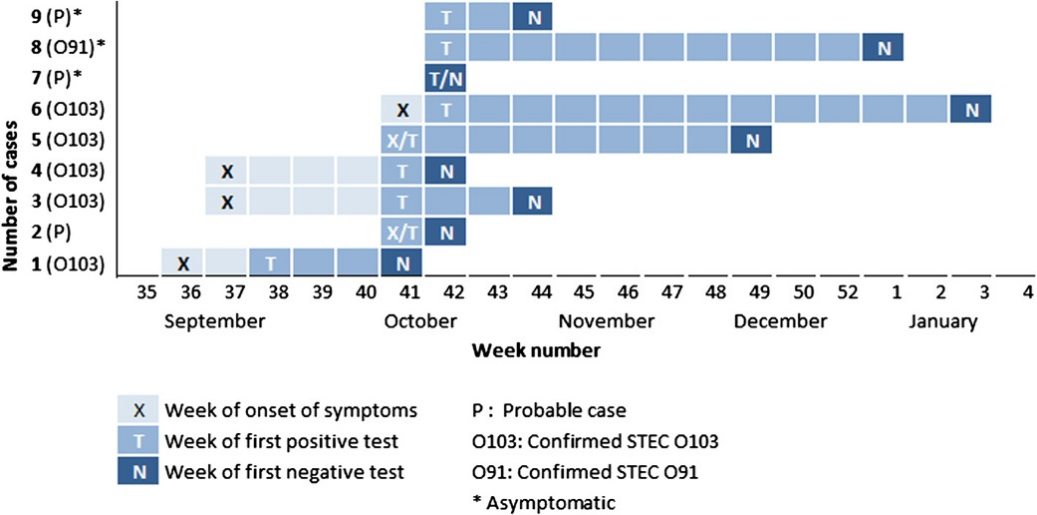
**Dates of symptom onset, first positive test and first negative test of confirmed and probable** ***E. coli*** **cases at the daycare, Norway 2012 – 2013.**

The median duration of shedding was 48 days for confirmed cases (range 30 – 98 days) (Figure 2). For probable cases, the median duration of shedding was 7 days (range 2 – 10 days). The median duration of exclusion from daycare for confirmed cases was 71 days (range 37 – 109 days). Most cases had consecutive negative test results once they had received the first negative results, and did not need to restart testing due to a positive result. However, two cases needed to restart the consecutive testing once. Three cases tested positive for EPEC after testing positive for STEC, which were considered negative results.

### Environmental investigation

The NFSA inspection found that the daycare centre had good food handling and hygiene routines in place. Food in the daycare facility is prepared and served by a trained food handler. A review of menus provided by the food handlers at the daycare centre revealed no suspicious food items. Temperature control practices were in place and food provision at the daycare was well organized. The daycare uses food items supplied by wholesale food distributers and local supermarkets. In addition, the designated areas for toileting and changing diapers were well maintained and had wash basins with hot water, soap dispensers and ethanol dispensers available at each station. The employees had all undergone hygiene training and the centre had well established routines.

## Discussion

This outbreak of *E. coli* infections reinforces that daycare centres are conducive to the spread of gastrointestinal illness among children. The source of the outbreak is unknown but it is likely that the STEC O103:H2 and STEC O91:H- serotypes were introduced to the daycare centre by separate index cases and then transmitted between the children, staff and family members. This is supported by symptom onset dates among confirmed cases being staggered over a two-month period. In addition, there were no indications from the trawling questionnaires or cohort investigation of any common sources of infection.

At the time of the described outbreak alert, it appeared that multiple children attending the daycare center could have STEC infections linked to STEC O103. Local health officials deemed it appropriate to close the daycare temporarily and screen all children. This control measure is not recommended for all daycare outbreaks but can be considered in severe circumstances. Ultimately no cases developed serious symptoms and the outbreak was interrupted, which may have been attributable to the implementation of this control measure. However, several children attending the daycare that were positive for STEC were asymptomatic. As screening can reveal unexpected results, the consequences of implementing screening must be thoroughly considered, particularly in terms of how children that are asymptomatic but positive for pathogenic *E. coli* will be managed.

The role of asymptomatic children in transmission of the infections in this outbreak is unknown. As several confirmed STEC cases were only identified through screening, it was not possible to determine the point at which they were infected, the duration of bacterial shedding prior to screening or whether the outbreak was propagated by asymptomatic shedders, particularly as the background level of diarrhea among children with negative STEC test results was high. Exclusion policies are based on the presumption that children can easily transmit STEC infection to other children when asymptomatic, and that ensuring children are no longer shedding the bacteria before they return to daycare will prevent spread of disease. However, the duration of the resulting exclusion period can be extensive, and keeping asymptomatic children home while waiting for multiple consecutive negative test results can have a substantial socioeconomic impact. Parents will often be required to miss work and children will be isolated from their peers for the duration of shedding, which has been reported to last up to 140 days [10]. In this case, nine children were excluded from daycare for a total of 459 days, with a median exclusion period of 53 days per child. This was longer than a previous Norwegian childcare outbreak of *E. coli* O145, where the median duration was 20 days (range 0-71 days). Although exclusion policies vary considerably from country to country, Norway has perhaps the most restrictive approach in the world with the recommendation to have up to five consecutive negative results before returning to daycare. These were developed in response to two recent serious outbreaks of STEC affecting children that lead to several HUS cases and at least one death [17].

Other Nordic countries [18], parts of Canada, the United States, Australia and the United Kingdom generally require two or three negative tests before returning to daycare. The difference between requiring two negative tests and five negative tests may not ultimately result in a substantially longer exclusion period, unless two negative tests are followed by a positive test. In this outbreak, the children that shed the bacteria for long periods of time were consistently positive until the first negative test, after which there was little variation between negative and positive test results. The difference between two and five tests only increased the exclusion period by 3 – 4 days, which is negligible after more than 100 days of positive tests. In this outbreak, specimens were initially tested by PCR and confirmed by culture. However, the increasing use of culture independent diagnostics may complicate the application of control measures, which in Norway are currently based on knowing the virulence profile and serogroup. As this information is not always available with PCR alone, more rigorous control measures than are necessary may be implemented pending further information and the time before the sufficient number of consecutive tests are negative may be extended. The need molecular characteristics information to inform appropriate control measures in outbreak situations should not be underestimated.

More research is needed regarding the risk of long-term asymptomatic shedders transmitting illness in different settings, including childcare facilities [19]. As further testing of the EPEC specimens was not conducted, it is unknown whether the EPEC infections found through screening are epidemiologically linked to the STEC cases. EPEC is also a cause of diarrhea, especially among children, but is also found in asymptomatic children and generally does not constitute a large problem in industrialized countries. EPEC has been notifiable in Norway since 1994, with more than 1200 cases reported between 1994 and 2011. Serotype O103:H2 has not been found in typical EPEC in Norway but has previously been found in five notified cases of atypical EPEC (aEPEC). For some serotypes, such as O26:H11, it has been shown that aEPEC and STEC are phylogenetically and genetically closely related and that they live in a dynamic relationship in which *stx* genes might be lost or gained [20],[21]. The substantial number of children positive for EPEC, and the children who were initially positive for STEC and subsequently positive for EPEC, may indicate that there was a relationship between STEC and EPEC in this outbreak, although this cannot be confirmed as analysis of the EPEC specimens was not conducted. However, the observed prevalence of aEPEC found through screening in a Norwegian daycare following an outbreak of STEC O145 in 2009 [7], and studies among healthy children and children with mild gastrointestinal symptoms indicate that aEPEC infection is frequently asymptomatic and that the endemic level of aEPEC is high in Norway [22],[23]. It is therefore not known whether the EPEC results identified through screening are attributable to isolates that have lost their *stx*1-encoding bacteriophage, a concomitant outbreak of EPEC, sporadic cases of different types of EPEC, or merely an expression of normal fecal flora.

The attack rate for confirmed and probable cases of STEC was highest among the groups for younger children, many of whom were still in diapers, while the outdoor group had no confirmed cases of STEC and the fewest children with either EPEC or reported diarrhea. The children in this group would have limited contact with other children attending the daycare, and therefore less likely to be infected through person-to-person contact. This reinforces that children who have gastrointestinal symptoms, particularly those still wearing diapers, may pose a greater risk to other children and staff members, than asymptomatic children who are carriers. Regardless of the etiology of gastrointestinal symptoms, children should be kept home for the recommended 48 hours following the cease of symptoms. Parents’ reports of duration of symptoms and number of days home from daycare in this outbreak suggest that children are not always kept home for a full 48 hours after symptoms subside, as is required by the daycare’s policy which is based on recommendations from the NIPH. Children that normally have loose stool are not required to stay home from daycare but it is the parents’ responsibility to assess whether a child has an unusual change in stool frequency or consistency. As the results of this investigation indicate that some parents find it challenging to determine when it is appropriate to keep children home from daycare, procedures at daycare centres should be reinforced regularly and parents should be encouraged to have a low threshold for keeping children at home. Following the outbreak, the daycare centre did clarify their protocols among staff and parents, but vigilance should be maintained during non-outbreak periods.

There are some limitations to this investigation. As a sensitive definition for possible cases of STEC infection was used, it is conceivable that cases of gastroenteritis of differing etiology, such as norovirus, occurred during the same period. This cannot be confirmed as except for the two initial cases of STEC, none of the children were tested for pathogens other than *E. coli*.

## Conclusions

In this outbreak, transmission of STEC infections most probably occurred through person-to-person contact, and there was no evidence of inappropriate food handling or food hygiene practices at the daycare centre. This outbreak shows the importance of having good routines in place for all children, not just those with obvious signs of illness, as one confirmed case and one probable case were asymptomatic and may have been shedding pathogenic *E. coli* bacteria without any signs of illness. It also highlights the confusion that may exist among parents as to what constitutes diarrhea in their children. While screening can identify asymptomatic cases, the implications should be carefully considered as it can produce unanticipated results and have significant socioeconomic consequences. Daycare exclusion policies should be reviewed to address the management of prolonged asymptomatic shedders and more research should be conducted on the effect of long-term asymptomatic shedders on transmission of STEC in daycare centres. We recommend heightening awareness among both staff and parents regarding the recommendation that children with gastrointestinal symptoms should be kept home for 48 hours after symptoms have subsided.

## Additional file

## Electronic supplementary material

Additional file 1: Web-based questionnaire for descriptive study at the daycare, October-November 2012 (in Norwegian). (PDF 492 KB)

Below are the links to the authors’ original submitted files for images.

Authors’ original file for figure 1

Authors’ original file for figure 2
