# Supplementary material for: Implications of screening and childcare exclusion policies for children with Shiga-toxin producing Escherichia coli infections: lessons learned from an outbreak in a daycare centre, Norway, 2012
Source: BMC Infect Dis. 2014 Dec 18;14:673. doi: 10.1186/s12879-014-0673-2 (PMC4279589; doi:10.1186/s12879-014-0673-2)
Supplement: Supplementary file 1 — Additional file 1: Web-based questionnaire for descriptive study at the daycare, October-November 2012 (in Norwegian). (PDF 492 KB) [file 12879_2014_673_MOESM1_ESM.pdf]

X barnehage

Flere barn ved X barnehage har vært syke med diare den siste måneden. Samtlige barn er derfor blitt testet for E.coli-infeksjon. For å prøve å finne ut hvorfor de har blitt syke har vi laget denne spørreundersøkelsen. Vi ber om at alle foreldre svarer på denne undersøkelsen en gang for hvert barn de har i X barnehage. Deltakelsen i spørreundersøkelsen er frivillig, men vi setter stor pris på om du kan sette av tid til å svare på spørsmålene under. Svarene behandles konfidensielt.

Det er viktig at både de som ble syke og de som ikke ble syke svarer på spørreskjemaet.

Vi vet det kan være vanskelig å huske tilbake, men selv om du ikke er 100% sikker kan du svare ut fra det du er ganske sikker på.

1) Barnets initialer

2) Kjønn

- Gutt
- Jente

3) Fødselsdato (dd-mm-åååå)

4) Barnehageavdeling

- A
- B
- C
- D
- E

5) Hvor mange voksne over 18 år er det i husstanden?

- 1
- 2
- 3
- 4
- Annet (spesifiser)

6) Hvor mange barn mellom 7 og 18 år er det i husstanden?

- 0
- 1
- 2
- 3
- 4
- Annet (spesifiser)

7) Hvor mange barn under 6 er det i husstanden?

- 1
- 2
- 3
- 4
- Annet (spesifiser)

8) Bruker barnet bleier?

- Ja
- Bare av og til
- Nei
- Annet (spesifier)

9) I hvilke situasjoner bruker barnet bleier? (Flere svar mulig)

- I barnehagen
- Om natten
- Ved lek ute
- Utenfør hjemmet (f. eks. på butikken)
- I andre situasjoner (spesifiser)

10) Når sluttet barnet med bleier?

11) Har barnet hatt diare, oppkast, eller magesmerter mellom 1. september og 17. oktober 2012?

- Ja
- Nei

12) Synes du det er vanskelig å avgjøre om barnet ditt har diare?

- Ja
- Nei

13) Før utbruddet, ved hvilke situasjoner ville du holdt barnet hjemme fra barnehagen? (Flere svar mulig)

- A) Hvis barnet har løsere og/eller hyppigere avføring en normalt
- B) Hvis barnet har mer enn 3 løse avføringer i løpet av 24 timer
- C) Hvis barnet kaster opp/har magesmerter
- D) Hvis barnet har A), B) eller C) OG i tillegg virker syk/slapp
- E) Hvis barnet A), B) eller C) OG i tillegg har feber
- F) Etter anbefaling fra lege/helsestasjon
- G) Annet (spesifiser)

14) Hvor mange perioder med diare, oppkast eller magesmerter hadde barnet mellom 1. september og 17. oktober 2012? Dersom barnet var syk flere dager sammenhengende telles det som 1 periode.

- 1
- 2
- 3
- 4
- 5+
- Annet

Dersom barnet ditt hadde diare, oppkast eller magesmerter mer enn en periode siden 1. september, gjelder de følgende spørsmålene *den siste gangen* barnet var sykt.

15) * Hvilken dato startet symptomene? (dd-mm-åååå)
16) Hvor lenge varte symptomene?

- En halv dag
- 1 dag
- 2-3 dager
- 4-5 dager
- 6-7 dager
- 1-2 uker
- Over 2 uker
- Er fremdeles syk

17) Hvilke symptomer hadde barnet? (Flere svar mulig)

- Hyppigere avføring
- Løsere avføring
- Diare
- Vanntynn diare
- Blod i avføring/blodig diare
- Forstoppelse
- Oppkast
- Kvalme
- Magesmerter/mageknip
- Nedsatt matlyst
- Slapp
- Feber
- Annet (spesifiser)
- Vet ikke

18) Var barnet hjemme fra barnehagen?

- Ja
- Nei

19) Eventuelt, hvor mange dager var barnet hjemme?

- Ingen
- 1
- 2-3
- 4-5
- Mer enn 1 uke
- Annet (spesifiser)

20) Er barnet friskt nå?

- Ja
- Nei
- Annet (spesifiser)

21) Hvis barnet hadde diare, oppkast eller magesmerter mer enn en periode siden 1. september, ønsker vi at du gir opplysninger om start, symptomer og varighet for hver enkelt av episodene, hvis mulig.

22) Hvor mange dager var barnet hjemme fra barnehage totalt i forbindelse med disse periodene?

23) Har barnet blitt testet for E. coli siden 1. september 2012?

- Ja
- Nei
- Vet ikke

24) Hvis ja, når ble den første prøven tatt? (dd-mm-åååå)

25) Hva var resultatet? (hvis det er tatt flere prøver hva var resultatet av den første?)

- Negativ

- Positiv for EPEC (den mindre alvorlige typen av E.coli-infeksjon)
- Positiv for EHEC (den mer alvorlige typen av E.coli-infeksjon)
- Har ikke fatt svar ennå
- Annet (spesifiser)
- Vet ikke

26) Hvorfor ble barnet testet?

- Fordi han/hun hadde diare, oppkast eller andre symptomer på tarminfeksjon
- Hadde ikke symptomer, men ble bedt av barnehagen å ta prøve
- Annet (spesifiser)

27) Ble barnet testet for annet enn E. coli?

- Ja
- Nei
- Vet ikke

28) Hvis ja, ble det påvist noen andre infeksjoner? Hvilke(n)?

29) Hadde andre i familien diare, oppkast eller magesmerter mellom 1. september og 17. oktober?

- Ja
- Nei
- Vet ikke

30) Hvis ja, hvem i familien ble syke?

- Mor
- Far

☐ Søsken

☐ Andre (spesifiser) [ ]

31) Hvis ja, hvilke symptomer hadde de og når ble de syke?

32) Ble de prøvetatt?

○ Ja

○ Nei

○ Vet ikke

33) Hva var resultatet?

34) Har barnet vært på et gårdsbruk i perioden 1. september til 17. oktober?

○ Ja

○ Nei

○ Vet ikke

35) Hvis ja, hvor og når?

36) Har barnet hatt kontakt med dyr (klappet, løftet, kost eller melket) mellom 1. september og 17. oktober? (Flere svar mulig)

☐ Nei

☐ Hund

☐ Katt

☐ Geit

☐ Hest

☐ Kanin

☐ Høne/hane

- [ ] Ku
- [ ] Sau
- [ ] Gris
- [ ] Andre dyr (spesifiser) ____

37) Har barnet smakt/drukket upasteurisert melk mellom 1. september og 17. oktober?

- ( ) Ja
- ( ) Nei
- ( ) Vet ikke

38) Var barnet utenlands i perioden 1. september til 17. oktober?

- ( ) Ja
- ( ) Nei

39) Hvis ja, hvor og når?

____

40) Har andre personer i barnets husholdning vært utenlands i løpet av den siste måneden før barnet ble syk?

- ( ) Ja
- ( ) Nei

41) Hvis ja, hvor og når?

____

42) Hvor får husholdningen vann fra?

- ( ) Kommunalt vannverk
- ( ) Privat vannforsyning (egen brønn e.l.)

43) Hvis privat, angi type.

____

44) Hva tror du selv er årsaken til sykdommen? Hvorfor?

45) Har du noe annet du vil formidle til oss?

© Copyright www.questback.com. All Rights Reserved.
