# Supplementary figures and images for: Implications of screening and childcare exclusion policies for children with Shiga-toxin producing Escherichia coli infections: lessons learned from an outbreak in a daycare centre, Norway, 2012
Source: BMC Infect Dis. 2014 Dec 18;14:673. doi: 10.1186/s12879-014-0673-2 (PMC4279589; doi:10.1186/s12879-014-0673-2)

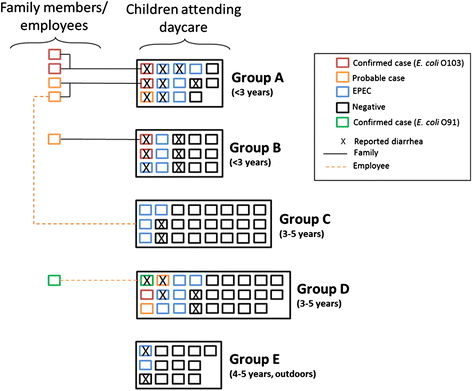

Supplement: Supplementary file 2 — Authors’ original file for figure 1 [file 12879_2014_673_MOESM2_ESM.gif]

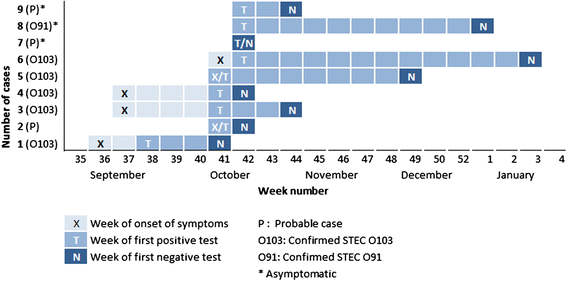

Supplement: Supplementary file 3 — Authors’ original file for figure 2 [file 12879_2014_673_MOESM3_ESM.gif]
